# Assessment of sub-maximal aerobic capacity in North African patients with chronic hepatitis B: a pilot case-control study

**DOI:** 10.12688/f1000research.160390.1

**Published:** 2025-01-16

**Authors:** Jihene Bergaoui, Imed Latiri, Sawssen MRAD, Houda Chaouch, Salma Amous, Jihene Ben Abdallah, Samia Ernez Hajri, Helmi Ben Saad

**Affiliations:** 1 Hospital Farhat HACHED, Research laboratory “Heart Failure, LR12SP09, Universite de Sousse Faculte de Medecine de Sousse, Sousse, Sousse, 4000, Tunisia; 2 Biochemistry Research Laboratory (LR18ES47), Farhat Hached University Hospital of Sousse, Sousse, Sousse, 4000, Tunisia; 3 Department of Infectious Diseases, Viral Hepatitis Research Unit (UR12SP35), Farhat Hached University Hospital of Sousse, Sousse, Sousse, 4000, Tunisia

**Keywords:** Aerobic Capacity; Aging; Chronic Disease; Exercise Test; Physical Fitness; Physical Intolerance

## Abstract

**Background:**

Studies assessing sub-maximal aerobic capacity in non-cirrhotic chronic hepatitis B (CHB) patients are scarce. This study aimed to evaluate sub-maximal aerobic capacity in CHB patients compared to apparently healthy participants (control-group).

**Methods:**

A 6-min walk test was performed. The 6-min walk distance (6MWD) was recorded, along with heart-rate, oxygen hemoglobin saturation (SpO _2_), blood pressure, and dyspnea ( ***ie***; visual analogue scale) at rest ( _Rest_) and at the end ( _End_) of the test. Additionally, 6-min walk work (6MWW), “6MWD × body mass index (BMI), “6MWD × SpO _2End_”, and “estimated cardiorespiratory and muscular chain age” were calculated. Signs of physical intolerance were determined including abnormal 6MWD ( ***ie***; 6MWD < lower limit of normal), chronotropic insufficiency ( ***ie***; heart-rate _End_ < 60% of maximal predicted heart-rate), high dyspnea ( ***ie***; dyspnea _End_ > 5), and desaturation ( ***ie***; drop in SpO _2_ > 5 points).

**Results:**

Compared to the control-group (n=28), the CHB-group (n=26) exhibited significantly lower 6MWD by 61 meters (13%), lower 6MWW by 5266 m.kg, lower “6MWD × BMI” by 1498 m.kg/m ^2^, lower “6MWD × SpO _2End_” by 5650%, and lower heart-rate _End_ by 26 bpm (12% predicted). The CHB-group included higher percentages of participants with chronotropic insufficiency (23.08% vs. 3.57% in the CG) and abnormal 6MWD (34.61% vs. 3.57% in the CG). CHB accelerated the aging of the “cardiorespiratory and muscular chain” by 11 years.

**Conclusion:**

Non-cirrhotic CHB may contribute to reduced submaximal aerobic capacity and acceleration of “cardiorespiratory and muscular chain” aging. A regular physical activity program could be a valuable intervention to mitigate these effects.

**Abbreviations’ list**
BMI: body mass index
CHB: chronic hepatitis B
COVID-19: coronavirus disease-19
CRMC: cardiorespiratory and muscular chain
DBP: diastolic blood pressure
ECRMC: estimated cardiorespiratory and muscular chain
_End_
: at the end of the 6-min walk test
HBsAg: hepatitis B surface antigen
HBV: hepatitis B virus
HR: heart-rate
MPHR: maximal predicted heart-rate
NC-CHB
: non-cirrhotic chronic hepatitis B

$\dot{V}O_{2}$

: oxygen consumption

$\dot{V}O_{2\text{peak}}$

: oxygen consumption at peak of exercise
OSAHS: obstructive sleep-apnea-hypopnea-syndrome
_rest_
: before the of the 6-min walk test
SBP: systolic blood pressure
SpO _2_
: oxy-hemoglobin saturation
VAS: visual analog scale
VHB: viral hepatitis B
∆: delta
6MWD: 6-min walk distance
6MWT: 6-min walk test
6MWW: 6-min walk work

## Introduction

Viral hepatitis B (VHB) induces significant morbidity and mortality in the general population. ^1^ VHB can progress to chronicity if it persists for more than six months and can induce systemic manifestations. ^1– 3^ In addition to hepatic conditions such as cirrhosis or hepatocellular carcinoma, chronic hepatitis B (CHB) causes extrahepatic effects, which can impact exercise capacity [ ***eg***; myocardial damage and alterations in pulmonary and muscle function] ^4– 8^ and significantly worsen the morbidity and mortality associated with CHB, ^2^ with potential social disadvantages. ^6, 8, 9^ Some studies have reported the harmful impacts of CHB on the main elements of the chain involved during adaptation to both maximal ( ***eg***; oxygen consumption ( $\dot{V}O_{2}$ )) ^9, 10^ and sub-maximal ( ***eg***; 6-min walk test (6MWT)) ^10, 11^ aerobic exercise, namely the cardiorespiratory and muscular chain (CRMC). ^3, 4, 8, 10^ First, it “seems” that the resting cardiovascular system of CHB patients is altered, with 3% of them having cardiovascular disease. ^12^ Second, CHB induces a decrease in resting lung function, such as the forced vital capacity and/or forced expiratory volume in one second. ^8^ Third, CHB causes weakness of respiratory muscles with low maximal inspiratory and expiratory pressures, ^8^ and can affect muscle fibres leading to muscle injuries. ^5, 13^

Exercise tolerance is commonly quantified through the measurement of $\dot{V}O_{2}$ during a cardiorespiratory test, which necessitates advanced and expensive equipment, highly skilled personnel for its operation, and substantial financial resources. ^14^ These limitations have led to the adoption of simpler assessments, such as the 6MWT. ^15, 16^ The latter offers several advantages including enhanced safety, ease of administration, closer alignment with everyday activities, cost-effectiveness, and ready implementation on a large scale. ^15, 16^ Over the past two decades ( ***ie***; 2004-2024), the 6MWT has been extensively utilized in assessing functional exercise performance across diverse populations, including those with pulmonary, cardiac, and neuromuscular diseases. ^17– 20^ Studies assessing CHB-related incapacity in terms of maximal and sub-maximal aerobic exercise impairment are scarce. ^9– 11^ As of late December 2024, it “seems” that only two studies have assessed $\dot{V}O_{2}$ in CHB patients, ^9, 10^ and only one Saudi study has compared the 6MWT data of CHB patients to those of healthy participants. ^11^ On the one hand, $\dot{V}O_{2}$ at peak of exercise ( $\dot{V}O_{2\text{peak}}$ ) was a predictor of mortality, as patients with a low $\dot{V}O_{2\text{peak}}$ ( ***ie***; < 17 ml/kg) had a survival rate of 55%, ^10^ and it is significantly correlated with maximal inspiratory pressure (r = 0.64) and with the Model for End-Stage Liver Disease (r = 0.91). ^9^ On the other hand, the authors of the Saudi study reported that compared to healthy participants (n=45), patients with CHB (n=49) had a significantly lower 6-min walk distance (6MWD) by 31 m. ^11^ The Saudi study had some methodological weaknesses that can “slightly” modify the findings. ^11^ First, the inclusion of patients with diverse liver diseases ( ***eg***; non-cirrhotic chronic hepatitis B (NC-CHB) or C, cirrhotic), is a source of ‘perplexity’ since the clinical outcomes are different. ^6^ Second, the absence of sample size determination is a statistical flaw. ^21^ Third, the expression of the main outcome ( ***ie***; 6MWD) only in absolute value, and the lack of its standardization according to participants’ characteristics ( ***eg***; sex and anthropometric data), could lead to misinterpretation. ^8, 10, 11^ The standardized 6MWD allows a more objective comparison between the diverse groups. ^22^ Fourth, the use on the quantitative significance approach with a “p value” < 0.05 is criticized, and the qualitative significance approach is recommended in medical exercise research. ^23^

To the finest of the authors’ knowledge, no previous study has explored the incapacity via the 6MWT in a homogeneous group of NC-CHB patients compared to “apparently” healthy participants. The main aim of this case-control study was to compare the 6MWT data of the two groups. The null hypothesis was that the two groups would have comparable 6MWD ( ***ie***; the main outcome).

## Methods

The present study is part of a larger project, involving two groups (CHB patients and “apparently” healthy controls) and comprising three parts. The project’s methodology was published as a “protocol in progress”. ^6^ The first part of the project evaluated muscle mass and strength in CHB patients. ^7^ The main conclusion was that NC-CHB does not affect muscle mass and strength. ^7^ The second part, which is the focus of this study, examines sub-maximal aerobic capacity. The third and fourth parts will evaluate the quality-of-life and oxidative status of the two aforementioned groups, respectively.

### Study design

This project is a case-control study conducted during the decline of the coronavirus disease (COVID-19) pandemic in Tunisia ( ***eg***; September 2020). The study was conducted in collaboration with the department of physiology at the faculty of medicine of Sousse (Sousse, Tunisia) and three departments from Farhat HACHED hospital in Sousse ( ***ie***; infectious diseases, biochemistry, and hematology). The study was conducted following the guidelines established by the STROBE statement. ^24^

Each participant received comprehensive information about the study’s objectives, procedures, potential risks, and other pertinent details. After this thorough briefing, we obtained written informed consent from all participants, confirming their voluntary involvement in the study. Additionally, each participant was provided with a report of his/her individual evaluations.

### Study population

**Figure 1.**
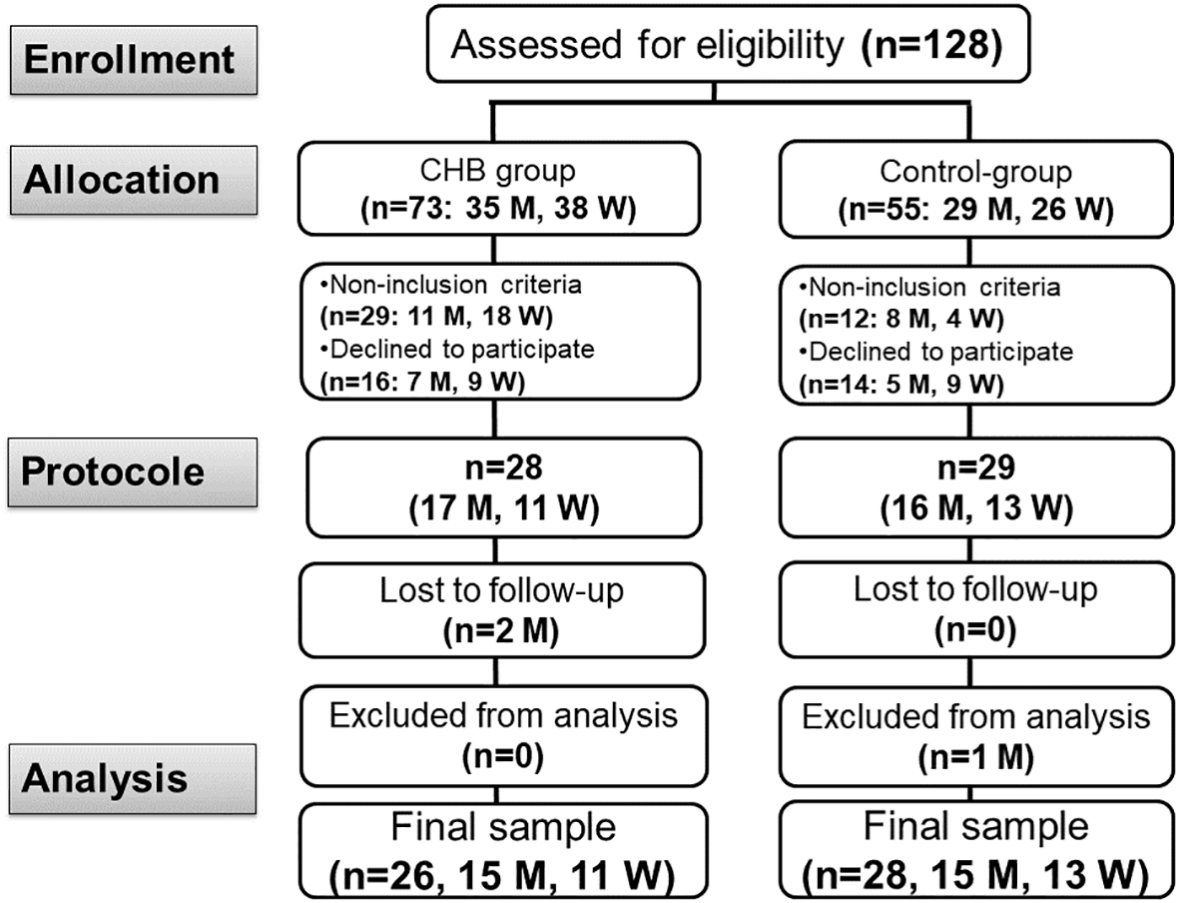
Study flow chart. CHB: chronic hepatitis B. M: man. W: woman.

Two groups of participants ( ***ie***; cases and controls) were recruited ( Figure 1).

Cases were selected from patients followed for VHB infection, who had undergone histological evaluation by liver biopsy (LB) or fibroscan within the last four years prior to inclusion in the study at the above infectious diseases department. Diagnosis of hepatitis B virus (HBV) infection was based on a positive result for hepatitis B surface antigen (HBsAg) for at least six months. Patients were eligible for inclusion if they were aged between 25 and 55 years, had an HBV-DNA viral load higher than 2000 IU/ml confirmed at least one year prior to inclusion, and showed no significant pathological fibrosis, as indicated by a fibroscan score less than 6 kPa and/or a “meta-analysis of histological data in viral hepatitis” score less than A2F2. Exclusion criteria were as follows: physical or mechanical impairments that could interfere with the 6MWT such as a history of orthopedic or rheumatologic diseases, contraindications for the 6MWT like signs of unstable angina or myocardial infarction within the previous month, resting heart-rate (HR) higher than 120 bpm, systolic blood pressure higher than 180 mmHg, or diastolic blood pressure hgher than 100 mmHg), ^16^ comorbidities such as respiratory or cardiovascular diseases, systemic conditions that could influence blood test results like diabetes mellitus or renal failure, consumption of alcohol, co-infection with other viruses, liver damage, or the requirement for CHB treatment during the study period.

Controls were “apparently” healthy participants, non-alcohol consumers, aged between 25 and 55 years, without any chronic disease, physical problems, COVID-19 infection, or 6MWT contraindications. ^16^

Participants from both groups with missing biological data were excluded from the final statistical analysis.

### Sample size

The sample size was calculated using this equation ^25^:

$$N=\left(\left(r+1\right){\left(Z_{\alpha /2}+Z_{1-\beta }\right)}^{2}\;s^{2}\right)/\left({\mathrm{rd}}^{2}\right);$$

where

• “ *N*” is the the required number of participants ( *N* = *n*_1_ + *n*_2_, such as *n*_1_ and *n*_2_ are the sample sizes for the case and control groups);
• “ *Z*_*α/2*_” is the normal deviate at a level of significance (1.96for a 5% level of significance);
• “ *Z*_*1-β*_” is the normal deviate at 1- *β*% power with *β*% of type II error (1.28 at 90% statistical power);
• “ *r*” (= *n*_1_/ *n*_2_) is the ratio of sample sizes required for the two groups ( *r* = 1 gives a 1:1 sample size distribution for the two groups); and
• “ *s*” and “ *d*” are the pooled standard-deviation (SD) and difference of 6MWD means between the two groups. These values were derived from a previous Saudi case-control study comparing the 6MWD of CHB patients to healthy participants. ^11^ The controls and cases had 6MWD means of 421 and 390 m, respectively, with a mean SD of 50 m.

Inserting these values into the predictive equation resulted in a total sample size of 54 participants (27 in each group). Assuming a 10% loss of biological data, a revised sample size of 60 participants was determined (60 = 54/(1-0.10)).

### Study protocol

The explorations were conducted indoor between 8:00 AM and 12:00 PM, with four participants examined per day, and an average time of 60 minutes per participant. The study protocol included the following steps:

• Signing of consent and completion of medical questionnaires.
• Collection of anthropometric data and blood samples.
• Bioelectrical impedance analysis.
• Consumption of a food snack of choice.
• Measurement of handgrip-strength.
• Performance of the 6MWT.

### Medical and physical activity questionnaire

A questionnaire comprising three parts was administered by one qualified examiner ( ***JB*** in the authors’ list). The mean duration of the questionnaire was approximately 20 minutes.

The first part was a standard medical questionnaire (widely used in the infectious diseases and physiology departments) aiming at collecting clinical and socioeconomic data. Questions were asked in Arabic. Clinical histories, such as previous hospitalization, comorbidities, and viral co-infections were recorded. Cigarettes smoking was evaluated in pack-years, and participants were classified into two groups (non-smoker: <5 pack-years; smoker ^3^: 5 pack-years). ^6^ Depending on alcohol consumption habits, participants were classified into two groups (consumer/non-consumer). ^6^ Socioeconomic-level was determined according to the participant’s profession, with two levels defined ( ***ie***; unfavorable and favorable). ^6^ Schooling level was arbitrarily defined as low and high. ^6^ Parity (the number of children born to a woman) was noted. Since the 2023 Tunisian global fertility rate was 2.09 children per woman, a parity greater than two was considered “high”. ^26, 27^

The second part of the questionnaire was linked to the physical-activity level, which was estimated using the Voorrips questionnaire. ^28^ This questionnaire is reproducible, and its score is positively related to the 24-hour measurement of the physical-activity as assessed by a pedometer. ^28^ While the Arabic version is not validated, it has been widely used in previous studies. ^29– 31^ This questionnaire contains 51 items assessing various scores, which are divided into three categories of physical-activity ( ***ie***; daily, sports, and leisure activities). The sum of the three scores represents the total physical-activity score. According to the total score, participants were divided into two groups: sedentary (score <9.42) and active (score ≥9.42). ^28^

The third part of the questionnaire is related to the evaluation of quality-of-life using the Chronic liver diseases questionnaire. ^32^ Data from this part will be analyzed in a subsequent study.

### Sex and anthropometric data

Age (in years) and sex and were documented for each participant. Height was measured in centimeters using a Siber Hegner ^®^ standing stadiometer, with participants standing upright, without shoes, heels together, and back straight. Weight (in kilograms), muscle mass (in percentage), and body fat (in percentage) were assessed using a Beurer BF-600 (Beurer GmbH, Germany) bioelectrical impedance analyzer in the morning after an overnight fast, with participants in a standing position. ^33^ Body mass index (BMI, kg/m ^2^) was determined. Participants corpulence status was categorized based on BMI as follows: underweight (BMI < 18.5 kg/m ^2^), normal weight (BMI: 18.5–24.9 kg/m ^2^), overweight (BMI: 25–29.9 kg/m ^2^), and obesity (BMI ≥ 30 kg/m ^2^). All measurements were conducted by a single qualified examiner ( ***JB*** in the authors’ list).

### Biological data

Some biological data ( ***eg***; hemoglobin, erythrocyte-sedimentation-rate, C-reactive-protein, interleukin-6, alkaline-phosphatase, alanine-aminotransferase, aspartate-aminotransferase, gamma-glutamyl-transpeptidase, total-bilirubin, non-conjugated bilirubin, ( ***ie***; antioxidant stress marker), ^35^ albumin ( ***ie***; antioxidant stress marker), ^36, 37^ and uric-acid ( ***ie***; oxidant-antioxidant balance marker) ^38^ were collected by a nurse. Some of these biological data and their technical aspects were detailed elsewhere. ^6, 7^

### Handgrip strength

Handgrip-strength, the technique and findings of which were detailed elsewhere, ^6, 7^ was performed by one qualified examiner ( ***JB*** in the authors’ list). For this study, the highest absolute handgrip-strength value (kg) between the two hands of each participant was retained.

### 6-min walk test

Sub-maximal aerobic capacity was evaluated using the 6MWT, supervised by one qualified examiner ( ***IL*** in the authors’ list). Participants were asked to wear comfortable clothing and appropriate footwear for walking, and to avoid strenuous exercise in the two hours preceding the 6MWT. ^16^ A single 6MWT was performed in a 40 m flat corridor indoor ( ***ie***; physiology department), which was marked every meter with start and end indicators. ^39^ Instructions given before the 6MWT followed the most updated guidelines, ^16, 39^ which included “walk as far as possible for 6 minutes”. Participants were informed that they could slow down, stop, rest as needed, and resume walking when able. ^16, 39^ They were instructed not to run under any circumstances, and no encouragement or walking aids were provided during the 6MWT. ^16, 39^ The remaining time was announced every minute ( ***eg***; you have × minutes left). ^16, 39^ The examiner did not walk with the participants to avoid influencing their walking speed.

The following 6MWT data were recorded: HR [bpm, % of maximal predicted HR (MPHR (bmp) = 208-0.7×Age (year))], ^40^ dyspnea (absolute value), blood-pressure (mmHg), oxy-hemoglobin saturation (SpO _2_, %), number of stops while walking, and 6MWD (m, %). Additional relative 6MWD indices, including the product between 6MWD and weight ( ***ie***; 6-min walk work (6MWW, m.kg)), ^41^ BMI (m.kg/m ^2^), muscle-mass (m.kg), body-fat (m.kg), and SpO _2End_ (%m) were calculated. HR, SpO _2_, blood-pressure, and dyspnea were measured while the participant was seated at rest ( _Rest_) and immediately at the end ( _End_) of the 6MWT. ^16, 39^ HR and SpO _2_ were measured using a handheld pulse oximeter (M700, Biolight CO., LTD. China), and blood-pressures were measured using a manual tensiometer and a stethoscope. Delta (∆) HR and SpO _2_ ( ***ie***; ∆HR (bpm) = HR _End_ – HR _Rest_, ∆SpO _2_ = SpO _2End_ - SpO _2Rest_) were calculated. Dyspnea was measured on a visual analog scale (VAS) ranging from 0 (no dyspnea) to 10 (maximum dyspnea). ^42^

The 6MWD was expressed both in absolute value (m) and as a percentage of the predicted 6MWD. ^22, 43^ For participants under 40 years of age, the following predictive equation for 6MWD specific to the North African population was applied: 6MWD (m) = 800.05-64.71 × Sex (0: Man; 1: Woman) - 10.23 × BMI (kg/m ^2^) - 1.63 × Age (years) + 2.05 × Weight (kg). ^43^ For this group, the lower limit of normal (LLN) was calculated by subtracting 74.31 m from the predicted 6MWD value. ^43^ For participants older than 40 years of age, the following predictive equation for 6MWD specific to the North African population was applied: 6MWD (m) = 720.50-160.27 × Sex (0: Man; 1: Woman) - 5.14 × Age (years) - 2.23 × Weight (kg) + 271.98 × Height (m). ^22^ For this group, the LLN was calculated by subtracting 89 m from the predicted 6MWD value. ^22^

Since the 6MWT assesses the integrated response of the CRMC, ^39, 44^ its estimated age (ECRMC) was calculated using the following formulas: ECRMC (years) = 184.25-0.36 × measured 6MWD (m) + 44.39 × Height (m) - 13.87 × Sex (0: Man; 1: Woman); for participants under 40 years of age ^45^; and ECRMC (years) = 140.17-0.19 × measured 6MWD (m) - 31.18 × Sex (0: Man; 1: Woman) - 0.43 × Weight (kg) + 52.91 × Height (m); for participants aged 40 years and more. ^45^

The following definitions were applied based on previous studies ^22, 46, 47^:

i) Signs of walking intolerance: abnormal 6MWD ( ***ie***; 6MWD < lower limit of normal), stopping while walking, high dyspnea ( ***ie***; dyspnea _End_ >5/10);
ii) Clinically significant desaturation: ∆SpO _2_ >5 points; and
iii) Chronotropic insufficiency: HR _End_ <60%.

### Statistical analysis

The distribution of quantitative data was analyzed using the Shapiro-Wilk W test. Data were expressed as means±SD (and 95% confidence intervals) when the normality test was met. If not, data were presented as medians (interquartile range). The Wilcoxon matched-pairs test was used to compare chronological and ECRMC ages within each group. Two significant approaches were applied: ***i)*** Quantitative (statistical) approach: The Mann-Whitney U and Chi-2 tests were used to compare quantitative and categorical data, respectively, between the two groups; and ***ii)*** Qualitative (clinical) approach: The percentages of participants with walking intolerance signs, desaturation, chronotropic insufficiency were compared using the 2-sided Chi-2 test. Hedge’s unbiased d value was used to measure the effect size of the main outcome (6MWD). ^48^ The effect size was described as small (≤0.2), medium (around 0.5), large (around 0.8), or very large ( ^3^1.30). ^48^

All statistical procedures were performed using. STATISTICA (data analysis software system, version 12. www.statsoft.com, RRID: SCR_014213). The significance level was set at p<0.05.

## Results

Out of the 128 participants assessed, data from 54 participants [26 cases (15 men) and 28 controls (15 men)] were retained for the final dataset ( Figure 1).

Table 1 presents the characteristics of the two groups of participants. Compared to the control-group, the CHB-group was ≈5 years older and had higher percentages of participants with low schooling-level, and unfavorable socioeconomic-level. The two groups had comparable handgrip-strength values and physical-activity scores, and included comparable percentages of smokers and sedentary participants. No participant consumed alcohol.

**Table 1.** Characteristics of the chronic hepatitis B (CHB, n=26) and control (CG, n=28) groups.

Data	Unit/category	CHB group	CG	Mean change	p-value
**Sex and anthropometric data**					
**Sex** ^**b**^	women	11 (42.31)	13 (46.43)	-	0.761
**Chronological age** ^**a**^	year	42±6 (40 to 45)	37±7 (34 to 39)	5	0.006 ^*^
**ECRMC age** ^**a**^	year	53±26 (43 to 64)	25±28 (14 to 36)	28	0.001 ^*^
**Delta age (Chronological age – ECRMC)** ^**a**^	year	-11±24 (-21 to -2) ^**#**^	12±25 (2 to 22) ^**#**^	-23	0.001 ^*^
**Age range < 40** ^**b**^	years	10 (38.46)	20 (71.43)	-	0.015 *
**Height** ^**a**^	cm	171±10 (167 to 175)	173±10 (169 to 176)	-2	0.822
**Weight** ^**a**^	kg	82±18 (74 to 89)	82±14 (76 to 87)	0	0.634
**Muscle mass** ^**a**^	%	34.4±7.6 (5.93 to 10.45)	34.7±7.1 (5.63 to 9.70)	0	0.883
**Body fat** ^**a**^	%	33±15 (11.43 to 20.13)	31±13 (9.91 to 17.07)	2	0.863
**Body mass index** ^**a**^	kg/m ^2^	27.8±5.8 (25.5 to 30.2)	27.5±4.1 (25.9 to 29.1)	0	0.979
**Corpulence status** ^**b**^	normal weight	9 (34.61)	7 (25.00)	-	0.728
	overweight	10 (38.46)	13 (46.42)	-	
	obese	7 (26.92)	8 (28.57)	-	
**Parity, habits, socioeconomic data**					
**Parity** ^**a**^		2±1 (1 to 2)	1±1 (1 to 2)	1	0.247
**High parity** ^**b**^	>2	1 (3.8)	1 (3.6)	-	0.969
**Smoker** ^**b**^	yes	10 (38.46)	5 (17.85)	-	0.091
**Schooling-level ** ^**b**^	low	8 (30.76)	1 (3.57)	-	0.007 ^*^
**Socioeconomic-level ** ^**b**^	unfavorable	9 (34.61)	3 (10.71)	-	0.035 ^*^
**Physical activity scores and levels**					
**Daily activities** ^**a**^		1.77±0.45 (1.58 to 1.95)	1.76±0.81 (1.45 to 2.08)	0	0.436
**Sports activities** ^**a**^		0.72±2.03 (-0.10 to 1.54)	2.13±3.88 (0.62 to 3.63)	-1.41	0.279
**Leisure activities** ^**a**^		0.75±1.63 (0.0 to 1.41)	0.38±0.84 (0.05 to 0.71)	0.37	0.653
**Total score** ^**a**^		3.24±3.18 (1.95 to 4.52)	4.27±4.45 (2.55 to 6.00)	1.03	0.616
**Sedentary** ^**b**^		25 (96.15)	25 (89.29)	-	
**Muscle function**					
**Handgrip strength (highest absolute value)** ^**a**^	kg	41±10 (8.07 to 14.2)	43±13 (10.64 to 18.33)	-2	0.697
**Viral charge, liver biopsy puncture and fribroscan score**					
**Viral charge** ^**c**^	IU/mL	5230 (3180-12786)	-	-	-
**Liver biopsy puncture** ^**α** **,** **b**^	A0F0	8 (30.77)	-	-	-
	A0F1	6 (23.07)	-	-	-
	A1F0	14 (53.85)	-	-	-
	A1F1	10 (38.46)	-	-	-
**Fibroscan score** ^**β** **,** **a**^	KPa	4.67±1.15	-	-	-

**A**: inflammatory activity ( **A0** = No histologic necroinflammatory activity, **A1** = Minimal activity). **ECRMC**: estimated cardiorespiratory and muscular chain. **F**: fibrosis staging ( **F0** = No fibrosis, **F1** = Portal fibrosis without septa). Mean change: (CHB group – CG).

Data were:

^a^ Mean±standard deviation (95% confidence interval);

^b^ Number (%);

^c^ Median (interquartile).

^*^ p-value < 0.05 (Mann-Whitney U test or 2-sided Chi-2): CHB group vs. CG.

^#^ p-value < 0.05 (Wilcoxon matched pairs test): Chronological age vs. ECRMC for each group.

^α^ Liver biopsy puncture was performed in 13 patients.

^β^ Fibroscan score was performed in 21 patients.

The CHB-group and the control-group had comparable values of hemoglobin (14.51±1.92 vs. 14.54±1.79 g/dL, respectively), erythrocyte-sedimentation-rate (6.96±7.13 vs. 7.36±6.95, respectively), CRP (5.39±0.98 vs. 5.93±1.54 mg/L, respectively), alkaline-phosphatase (50.61±16.60 vs. 41.64±16.07 UI/L, respectively), alanine-aminotransferase (16.26±6.58 vs. 16.89±11.54 UI/L, respectively), aspartate-aminotransferase (21.77±5.09 vs. 20.89±13.02 UI/L, respectively), gamma-glutamyl-transpeptidase (15.54±9.55 vs. 15.46±10.76 UI/L, respectively), non-conjugated bilirubin (12±16 vs. 9±5 μmol/L, respectively), total-bilirubin (14±16 vs. 10±5 μmol/L, respectively), albumin (44±3 vs. 44±3 g/L, respectively), uric acid (258±82 vs. 253±60 μmol/L, respectively), and interleukin-6 (1.7±0.9 vs. 1.9±1.7, respectively.

**Figure 2.**
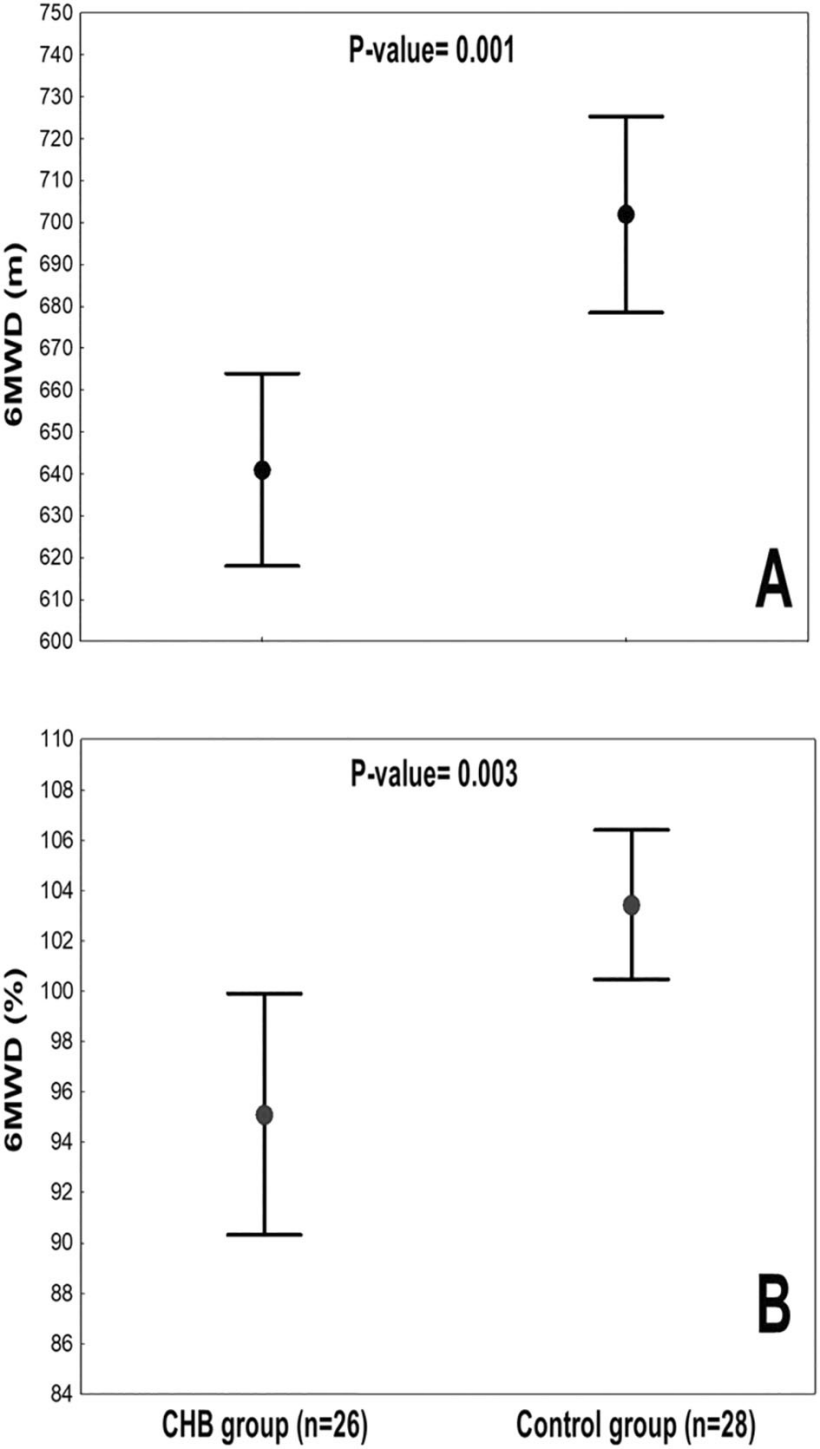
6-min walk distance (6MWD) values of the 2 groups: chronic hepatitis B (CHB) group and control-group. A. 6MWD expressed as absolute value (m) B. 6MWD expressed as percentage of predicted values. Data were mean (●) and 95% confidence interval ( 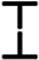).

Table 2 presents the 6MWD data and its relative indices, and Figure 2 exposes the 6MWD values for the two groups. Regarding the 6MWD, compared to the control-group, the CHB-group covered a statistically significantly shorter distance by ≈61 m (702±60 vs. 641±57 m, respectively) ( Figure 2A, Table 2) and by ≈8% (103±8 vs. 95±12%, respectively ( Figure 2B, Table 2). The Hedge’s unbiased d for the 6MWD (m, %) were small at -1.026 and -0.980, respectively. In terms of the relative 6MWD indices, compared to the control-group, the CHB-group had statistically lower 6MWW by 5266 m.kg, “6MWDxBMI” by 1498 m.kg/m ^2^, and “6MWDxSpO _2End_” by 5650 %m. The “6MWDxmuscle-mass” and “6MWDxbody-fat” were comparable between the two groups.

**Table 2.** 6-min walk distance (6MWD) data and relative 6MWD indices of the chronic hepatitis B (CHB, n=26) and control (CG, n=28) groups.

Data	Unit	CHB group	CG	Mean change	p-value
**6MWD data**	m	641±57 (618 to 664)	702±60 (678 to 725)	-61	0.001 ^*^
	%	95±12 (90 to 100)	103±8 (100 to 106)	-8	0.003 ^*^
**6MWW**	m.kg	52193.81±12001.30 (9412.107 to 16566.70)	57459.03±11120.54 (8792.120 to 15136.58)	-5266	0.070 ^*^
**6MWD × BMI**	m.kg/m ^2^	17710±3344 (16359 to 19061)	19208±2860 (18100 to 20317)	-1498	0.056 ^*^
**6MWD × MM**	m.kg	22275±6005 (19850 to 24701)	24629±6424 (22138 to 27120)	-2354	0.143
**6MWD × BF**	m.kg	20537±8217 (17218 to 23856)	21550±7445 (18663 to 24437)	-1013	0.337
**6MWD × SpO**_**2End**_	%m	63070±5694 (60771 to 65370)	68720±6088 (66359 to 71081)	-5650	0.003 ^*^

**BF**: body fat. **BMI**: body mass index. **MM**: muscle mass. **SpO**_**2End**_: oxy-hemoglobin saturation at the end of exercise. **6MWW**: 6-min walk work. **Mean change**: (CHB group – CG).

Data were mean±standard deviation (95% confidence interval).

* p-value < 0.05 (Mann-Whitney U test or 2-sided Chi-2): CHB group vs. CG.

Compared to the control-group, the CHB-group had statistically lower HR _End_ by 26 bpm and 12% of MPHR, and DHR by 26%, and included a higher percentage of participants with chronotropic insufficiency ( Table 3).

**Table 3.** Heart rate (HR) data of the chronic hepatitis B (CHB, n=26) and control (CG, n=28) groups.

Data	Unit	CHB group	CG	Mean change	p-value
**HR**_**Rest**_^**a**^	bmp	67.5±7.9 (64 to 71)	68.1±10.8 (64 to 72)	1	0.843
	%	38±4 (36 to 40)	37±6 (35 to 40)	1	0.737
**HR**_**End**_^**a**^	bpm	127.1±26.6 (116 to 138)	153.2±22.2 (145 to162)	-26	0.001 ^*^
	%	71±14 (65 to 77)	83±13 (78 to 88)	-12	0.005 ^*^
**DHR** (= HR _End_ - HR _Rest_) ^**a**^	bmp	59.6±22.9 (50 to 68)	85.1±23.1 (76 to 94)	-26	0.001 ^*^
**Low HR** (HR _End_ < 60%) ^**b**^	-	6 (23.08)	1 (3.57)	-	0.033 ^*^

_**End**_: at the end of the 6-min walk test (6MWT). _**Rest**_: before the 6MWT. **Mean change**: (CHB group – CG).

Data were:

^a^ Mean±standard deviation (95% confidence interval) .

^b^ Number (%).

^*^ p-value < 0.05 (Mann-Whitney U test or 2-sided Chi-2): CHB group vs. CG.

The two groups had comparable blood-pressure, SpO _2_, and dyspnea, and included comparable percentages of participants with desaturation and high dyspena _End_ ( Table 4). Compared to the control-group, the CHB-group had a statistically higher DSpO _2_ by 1% and included a significantly higher percentage of participants with an abnormal 6MWD ( Table 4). All participants completed the 6MWT and none stopped during the test.

**Table 4.** Blood pressure, oxy-hemoglobin saturation (SpO _2_), dyspnea, and exercise intolerance’ signs of the chronic hepatitis B (CHB, n=26) and control (CG, n=28) groups.

Data (unit)	Phase or applied definitions	CHB group	CG	Mean change	p-value
**Blood pressure, SpO**_**2**_ **, and dyspnea**					
**SBP** (mmHg) ^**a**^	Rest	119±13 (114 to 125)	114±12 (109 to119)	5	0.130
	End	148±21 (139 to 156)	147±20 (139 to154)	1	0.972
**DBP** (mmHg) ^**a**^	Rest	77±11 (72 to 82)	73±11 (69 to 78)	4	0.257
	End	79±9 (75 to 83)	77±13 (72 to 82)	2	0.341
**SpO**_**2**_ (%) ^**a**^	Rest	97.9±0.9 (97.5 to 98.3)	98.3±0.8 (97.9 to 98.6)	-1	0.188
	End	98.4±0.9 (98.0 to 98.7)	97.9±1.2 (97.4 to 98.4)	1	0.177
**VAS dyspnea** ^**a**^	Rest	0	0	-	-
	End	1.6±1.2 (1 to 2)	1.1±0.8 (1 to 1)	0.5	0.156
**Exercise intolerance’ signs**					
**DSpO**_**2**_^**a**^	SpO _2End_ - SpO _2Rest_	0.5±1,2 (0.03 to 1.17)	-0.4±1.4 (-0.89 to 1.39)	1	**0.007** ^*^
**Desaturation** ^**b**^	DSpO _2_ > 5 points ^**b**^	0	0	-	-
**High dyspnea**_**End**_^**b**^	Dyspnea _End_ > 5 ^**b**^	0	0	-	-
**Abnormal 6MWD** ^**b**^	6MWD < LLN ^**b**^	9 (34.61)	1 (3.57)	-	**0.003** ^*^

**DBP**: diastolic blood pressure. _**End**_: at the end of the 6-min walk test (6MWT). **LLN**: lower limit of normal. _**Rest**_: before the 6MWT. **SBP**: systolic blood pressure **VAS**: visual analogue scale. **6MWD**: 6-min walk distance. **Mean change**: (CHB group – CG).

Data were:

^a^ Mean±standard deviation (95% confidence interval).

^b^ Number (%).

^*^ p-value < 0.05 (Mann-Whitney U test or 2-sided Chi-2): CHB group vs. CG.

Compared to the control-group, the CHB-group had a higher ECRMC age by 28 years (25±28 vs. 53±26 years, respectively) ( Table 1). The comparison of chronological and ECRMC ages revealed accelerated aging of the CRMC by 11 years in the CHB-group, and decelerated aging in the control-group by 12 years ( Table 1).

## Discussion

This case-control study reveals that CHB impacts sub-maximal aerobic capacity. Specifically, the CHB-group demonstrated statistically significant reductions in several parameters compared to the control-group: a decrease of 61 m (13%) in the 6MWD, lower 6MWW by 5266 m.kg, lower “6MWDxBMI” by 1498 m.kg/m ^2^, lower “6MWDxSpO _2End_” by 5650 %m, and lower HR _End_ by 26 bpm and 12% of MPHR. Additionally, the CHB-group had higher percentages of participants with chronotropic insufficiency (23.08% vs. 3.57%) and abnormal 6MWD (30.76% vs. 3.57%). Consequently, the null hypothesis that the two groups would have comparable 6MWD was rejected. Furthermore, was found to accelerate the aging of the CRMC by 11 years.

As of late 2024, and to the best of the authors’ knowledge, only one Saudi study, ^11^ detailed in **Appendix 1** has compared 6MWT data of patients with hepatic pathologies, including 49 CHB patients, to those of a control-group.

### Discussion of results

The CHB-group had a lower 6MWD by approximately 61 m compared to the control-group, aligning with the Saudi study, ^11^ which reported a difference of about 41 m. Additionally, the CHB-group exhibited an approximately 8% lower 6MWD expressed as a percent of predicted value **(Appendix 1)**. No previous study have expressed the 6MWD as a percent of a predicted value. Relative 6MWD indices in CHB patients have not been previously evaluated. Compared to the control group, the CHB group had lower 6MWW by ≈5266 m.kg, “6MWDxBMI” by ≈1498 m.kg/m ^2^, and “6MWDxSpO _2End_” by ≈5650 %m, while “6MWDxmuscle-mass” and “6MWDxbody-fat” values were comparable between the groups. There are no prior case-control studies evaluating these indices in chronic disease patients. The 6MWW, reflecting the work done during the 6MWT, has been assessed in conditions such as HIV infection and chronic obstructive pulmonary disease, which reported lower 6MWWs in chronic patient. ^49– 51^

No previous studies have compared HR, SpO _2_, blood-pressure, and dyspnea data between CHB and control groups. Compared to the control-group, the CHB-group had lower HR _End_ (bpm and %PMHR) by ≈26 and ≈12, respectively, and ΔHR by ≈26 bpm, with a higher percentage of patients exhibiting chronotropic insufficiency (4% vs. 23%) **(Appendix 1)**. Despite being “apparently” free from cardiovascular diseases, chronotropic insufficiency in CHB patients could be a preclinical sign of incipient cardiovascular pathology, as 3% of CHB patients are reported to develop cardiovascular diseases. ^12^

Compared to the control-group, the CHB-group had a higher ΔSpO _2_, but comparable SpO _2Rest_ and SpO _2End_ **(Appendix 1)**. The higher ΔSpO _2_ observed in our CHB-group lacks clinical significance as both groups had comparable SpO _2_ values, and no participant experienced “clinically significant desaturation”. This suggests that the alveolo-capillary membrane remains intact.

Both groups had comparable blood-pressure and VAS dyspnea **(Appendix 1)**, indicating that CHB does not significantly affect blood-pressure or dyspnea.

Compared to the control-group, the CHB-group had a higher ECRMC’ age by ≈23 years ( Table 1). The ECRMC age for the CHB and control groups was higher by ≈11 years and lower by ≈12 years compared to chronological age, respectively. This indicates accelerated CRMC aging, similar to findings reported in diabetic patients. ^52^

*Factors explaining the decline in 6MWD and acceleration of CRMC aging in NC-CHB patients*

Several factors may explain the decline in 6MWD and the acceleration of CRMC aging in NC-CHB patients, including comorbidities ( ***eg***; cardiac, respiratory, and/or muscular diseases), patient characteristics ( ***eg***; age, corpulence status, schooling-level, socioeconomic-level, sedentarily lifestyle), parity, and smoking habits.

Chronotropic insufficiency may partly explain the 6MWD decrease, as seen in previous studies involving obstructive sleep apnea-hypopnea-syndrome (OSAHS) patients, ^45^ diabetic patients, ^52^ or narghile-smokers. ^31^ Although the impact of CHB on sinus node activity during walking was not documented, it affects sinoatrial node function. ^53^ Concerning the respiratory system, possible explanations for the 6MWD decrease include alterations in the alveolo-capillary membrane, bronchial airway, and respiratory muscle strength. The absence of SpO _2_ alterations suggests that the alveolo-capillary membrane is intact ( Table 4). However, lung function data alteration and respiratory muscle weakness indicate that these factors may contribute to the 6MWD decrease. ^8^ Muscle function was not a factor in our study, as both groups had comparable muscle-mass and handgrip-strength ( Table 1). Previous studies have reported no impairment in muscle strength in CHB patients, ^54, 55^ but handgrip-strength is a strong predictor of 6MWD, ^56^ and CHB can cause muscle injuries. ^5, 13^

The 5-year age gap between the CHB and control groups is unlikely to account for the differences in 6MWD and HR, as adjustments were made for age. Literature presents conflicting results regarding the effect of age on 6MWD. ^22, 31, 45, 52^ While age has been identified as an independent predictor of 6MWD in diabetic patients, ^52^ it is a non-independent predictor in OSAHS patients ^45^ and narghile-smokers. ^31^ In healthy adults, age is a dependent predictor of 6MWD. ^22^

The corpulence status was not a factor in our study, as the two groups had comparable BMI values and comparable corpulence statuses ( Table 1). Literature also provides conflicting conclusions about the effect of BMI and corpulence status on 6MWD. ^22, 31, 45, 52^ While some studies consider BMI an independent predictor of 6MWD in OSAHS patients, ^45^ narghile-smokers, ^31^ and healthy adults, ^22^ others do not. ^52^ While some studies consider corpulence status an independent 6MWD predictor in diabetic patients, ^52^ others do not in OSAHS patients, ^45^ narghile-smokers, ^31^ and healthy adults. ^22^

In our study, the CHB-group encompassed higher percentages of participants with lower schooling-level and unfavorable socioeconomic-level ( Table 1). The effect of schooling-level and socioeconomic-level on 6MWD is also controversial in literature. ^22, 31, 52, 56, 57^ Schooling-level was an independent predictor in some studies of healthy adults, ^57^ but not in others. ^22, 31, 45, 52, 56^ Socioeconomic-level was an independent predictor in healthy adults ^22^ and diabetic patients, ^52^ but not in others including OSAHS patients ^45^ or narghile-smokers. ^31^ In, the socioeconomic-level was identified as a dependent 6MWD predictor.

Since the two groups were matched for physical-activity ( Table 1), physical-activity levels cannot explain the 6MWD decrease. Literature on physical-activity’s effect on 6MWD is mixed. ^22, 31, 45, 52^ While one study considered physical-activity an independent 6MWD predictor in diabetic patients, ^52^ two others considered it a non-independent 6MWD predictor in OSAHS patients ^45^ or narghile-smokers. ^31^ In healthy adults, ^22^ the physical-activity level was identified as a dependent 6MWD predictor.

Since the women of both groups were matched for parity data ( Table 1), the latter cannot explain the 6MWD decrease. In literature, parity is recognized as a 6MWD influencing factor in healthy adults, ^22, 58^ and patients with chronic conditions. ^45, 52^

The matched smoking status ( Table 1), also eliminates smoking as a cause for the 6MWD decrease, with conflicting literature results, ^45, 52^ While one study considered it an independent 6MWD predictor in diabetic patients, ^52^ but not in others including OSAHS patients. ^45^

*Pathophysiological mechanisms explaining 6MWD decline and CRMC aging acceleration*

Several mechanisms may explain the 6MWD decline and CRMC aging acceleration in NC-CHB patients, including anemia, inflammation, liver dysfunction, oxidative stress, and apoptosis. ^59– 69^

Anemia, ^59^ inflammation, ^60, 61^ and certain liver function markers (aspartate-aminotransferase, alkaline-phosphatase, and bilirubin) ^62, 63^ are associated with exercise capacity. Nonetheless, since both groups were matched for hemoglobin, inflammation, and liver function data, these factors alone do not fully explain the 6MWD decline.

CHB interferes with apoptosis signaling pathways, ^64, 65^ and oxidative stress may contribute to liver disease progression in CHB patients. ^66– 68^ Similar to chronic obstructive pulmonary disease, apoptosis in the quadriceps of CHB patients might impair muscle function, ^69^ while oxidative stress could affect functional capacity. ^30, 60^ Although albumin, non-conjugated bilirubin, and uric-acid values were comparable between groups, suggesting maintained oxidant-antioxidant balance, the oxidative stress factor cannot be completely ruled out in explaining the 6MWD decline.

### Discussion of methods

Several methodological points including, which may influence our results, require discussion. The following paragraphs will discuss the sample and effect sizes, participants’ characteristics, statistical analysis approaches, recruitment methods, 6MWT practice, and data collection.

***Sample and effect sizes***

In contrast to the Saudi study, ^11^ we calculated both sample and effect sizes. Determining an adequate sample size is crucial for ensuring sufficient power to detect statistical effects. ^70^ The effect size provides a quantitative measure of the strength and magnitude of the observed association between exposure and outcome variables. ^48^ Unlike p-values, which indicate only whether an association is statistically significant, the effect size offers a more comprehensive understanding of the practical significance of the relationship. ^48^ Our calculated sample size ( ***ie***; CHB-group = 26, control-group = 28) was smaller than that of the Saudi study ( ***ie***; CHB-group = 49, control-group = 45), ^11^ and the effect size for the 6MWD was small.

***Participants’ characteristics***

Several factors can influence the 6MWD, including anthropometric data ( ***eg***; age, height, weight, BMI, corpulence status, and muscle-mass), sex, biological data ( ***eg***; hematological, inflammatory, and biochemical data), parity, schooling-level, socioeconomic-level, physical-activity level, and muscle strength. The influence of these factors will be discussed below.

Age, height, weight, BMI, corpulence status, muscle-mass, and sex are known independent predictors of 6MWD. ^71– 74^ Compared to our study, the Saudi study ^11^ included participants with a broader age range (25-55 vs. 18-80 years), which could introduce confusion, as 6MWD is negatively correlated with age. ^71– 73^ In our study, the control-group was younger than the CHB-group by ≈5 years. To account for this, we applied two corrective actions. We used North African 6MWD reference equations to standardize 6MWD by age, ^22, 43^ and we expressed HR as a percentage of MPHR, accounting for age. ^40^ Anthropometric data ( ***ie***; height, weight, BMI, corpulence status, and muscle-mass), and sex were comparable between our two groups. The Saudi study ^11^ reported comparable age, height, and weight but did not compare BMI or corpulence status **(Appendix 1)**. This omission could lead to misinterpretation, as high BMI is associated with reduced functional capacity ^75^ and 6MWD. ^22^ Additionally, muscle-mass, an important factor influencing 6MWD ^74^ was comparable between groups. As done by Alameri et al., ^11^ comparable percentages of men and women were included in our study. Sex also influences 6MWT results, with women generally showing lower 6MWD than men. ^76^

The two groups were matched for all biological data. Our sample of CHB patients represents a real-life cohort. For instance, the mean hemoglobin value in our study (14.51±1.92 g/dL) is similar to that reported by Alameri et al. ^11^ (12.87±4.41 g/dL).

We reported data on parity, which was comparable between the two groups, with no women having high parity. Parity negatively correlates with 6MWD, with multiparous women showing lower 6MWD compared to nulliparous women. ^22^ This effect may be due to hormonal changes, biochemical modifications, or respiratory muscle impairment. ^22^

We reported schooling-level and socioeconomic-level data for our participants ( Table 1). The unfavorable socioeconomic-level among our NC-CHB patients reflects findings in African CHB patient. ^77^

We assessed physical-activity level and handgrip-strength, finding comparable data between the two groups. In our study 96% of the CHB-group had a sedentarily status ( Table 1), which aligns with a study reporting 60% of CHB patients as sedentary. ^78^ Reduced physical-activity often leads to altered muscle metabolism, decreased muscle-mass, and reduced physical capacity. ^74^ Handgrip-strength is a strong, independent predictor of 6MWD. ^56^

While the Saudi study. ^11^ employed only a quantitative approach to compare measured data, our study utilized both quantitative and qualitative approaches. The qualitative approach, such as comparing percentages of patients with abnormal 6MWD, is recommended in medical exercise research. ^23^

***Recruitment methods of the two groups***

Like what has been done by Alameri et al., ^11^ our CHB patients were recruited from those followed at outpatient clinics. The main limitation of such method is the potential for selection bias. ^79^ Outpatient clinics typically serve individuals with less severe or milder forms of illness compared to those admitted to hospitals. ^80^ This bias may affect the generalizability of the results to the broader population, including those not seeking regular medical care or those treated in other healthcare settings. ^79^

Contrary to the Saudi study, ^11^ where healthy participants were recruited from hospital employees and medical students, in our study, the “apparently” healthy participants were recruited from relatives of CHB patient and from the announcement on social media account. On the one hand, the method applied by Alameri et al. ^11^ may consist of more highly educated individuals with a higher socioeconomic-levels, which are more likely to come from the broader general population. On the other hand, some relatives of CHB patients could be unknowingly carrying HBV.

***6MWT practice***

Since the information about 6MWT practice and data collection during the test allow better interpretation and comparison of results among different studies, many details such as applied guidelines, corridor length, place, number of tests, day-time, encouragement and walking aids during the 6MWT, and number of investigators need to be discussed. First, as done in Saudi study, ^11^ we applied the most updated available guidelines ( ***ie***; 2002, ^39^ and 2014 guidelines, respectively). ^15, 16^ Using updated guidelines in medical research lies in the promotion of scientific rigor, patient safety, relevance, consistency, regulatory compliance, and improved clinical decision-making. Second, as done in Saudi study, ^11^ we reported the corridor’ length ( ***ie***; 30 and 40 m, respectively). The corridor length is essential for precise comparisons of 6MWT results ^81^ and can influence performance. ^15, 39^ Although it was recommended that the walking course must be 30 m in length, ^39^ research has shown that there are no significant differences in outcomes when tracks of lengths ranging from 15 to 50 m are used. ^81^ Third, unlike the Saudi study, ^11^ we reported the 6MWT practice place ( ***ie***; indoor as recommended). ^39^ Research has indicated that there is little difference in 6MWD ( ***ie***; mean difference 4 m) between indoor and outdoor courses. ^82^ Fourth, as done by Alameri et al. ^11^ we performed only one 6MWT. Although repeated testing is recommended to account for the familiarization effect on 6MWD, ^22, 71, 73, 83– 86^ the 6MWT is more appropriate for clinical settings, where the test is typically performed once. ^87^ Fifth, contrary to the Saudi study, ^11^ we reported the day-time of the 6MWT ( ***ie***; between 8 and 11 am). ^22, 84^ This period is characterized by a stable ambient temperature and humidity which can minimize the intraday effects. ^88^ Intraday variability can be a source of biased data. ^39^ Sixth, contrary to the Saudi study, ^11^ we mentioned that no encouragement or walking aids during the 6MWT was given to participants. The latter can influence the 6MWD. ^15, 16, 89, 90^ Finally, contrarily to the Saudi study, ^11^ where 6MWT was supervised by several investigators, only one investigator was implicated in our study. In patients with chronic conditions, the 6MWT data can be compared when conducted by different investigators. ^91^

### Collected data

Similar to the Saudi study, ^11^ we reported the main outcome of the 6MWT ( ***ie***; 6MWD) **(Appendix 1)**. Contrary to the Saudi study, we reported additional 6MWT data **(Appendix 1)**.

The 6MWT main outcome, which is the 6MWD, is reported frequently in meters, ^15, 16^ which could be a source of misinterpretation. ^8, 10, 11^ In our study, 6MWD was expressed as percentage of predicted 6MWD, and the LLN was calculated from predicted values. Comparing measured 6MWD to predicted values derived from norms is an important point since norms are essential to guide the diagnostic and prognostic use of the 6MWT and the success in medical decision-making depends as much on selecting and properly using norms and their limits. ^15, 39^

In our study, we reported some 6MWT secondary outcomes, including HR, SpO _2_, BP, dyspnea, ECRMC’ age, and 6MWD relative indices. The following sentences will discuss their clinical importance. First, HR was reported in pbm and as % of MPHR in order to avoid the age effect, and ΔHR was calculated. Expressing HR as % of MPHR accommodates individual variations in fitness levels and age, allowing for personalized exercise intensity assessment. ^41^ ΔHR calculation is essential since it correlated with 6MWD. ^86, 92, 93^ Second, SpO _2_ and ΔSpO _2_ were reported. Oxygen desaturation during a 6MWT provides information regarding exercise-induced desaturation, disease severity and disease progress. ^15, 16^ Third, BP was mentioned. Measuring BP during the 6MWT is valuable for assessing cardiovascular health, exercise responses, and overall patient safety. ^22, 45, 94^ It can aid in diagnosis, risk assessment, exercise prescription, and patient care in cardiovascular conditions. ^22, 45, 94^ Fourth, dyspnea was evaluated. On the one hand, dyspnea is a clinical sign of walking intolerance. ^22, 46, 47^ On the other hand, it reflects both the physiology of exercise limitation, and the impact of exercise limitation on daily life. ^15, 16^ Fifth, we estimated the ECRMC’ age, which reflects acceleration of ageing. ^39, 44^ Finally, we calculated some 6MWD relative indices ( ***eg***; 6MWW, “6MWDxBMI”, “6MWDxmuscle-mass”, “6MWDxbody-fat” and “6MWDxSpO _2End_”) in order to better estimate the work required to perform the 6MWT than 6MWD alone. ^41^ For example, since weight directly affects the energy required to complete the 6MWT, the 6MWW can offer valuable insights into patients’ functional capacity. ^15, 16^ The 6MWW correlates strongly with $\dot{V}O_{2\text{peak}}$ and is suggested as a parameter of patients’ fitness evaluation when gas exchange measurements are unavailable. ^95^ In addition, as weight is not the only and best tool reflecting the body composition, and as the adiposity has a key position in the beginning of inability, ^75^ we had multiplied the 6MWD by BMI, muscle-mass and body-fat. Moreover, since a positive and significant correlation was observed between 6MWD and SpO _2End,_^96^ we had multiplied the 6MWD by the SpO _2End._

***Study limitations***

This study has two major limitations. First, the “apparently” healthy group did not undergo HBV-DNA testing or fibroscan to confirm the absence of HBV infection and hepatic fibrosis, respectively. These tests were challenging to perform due to economic constraints and ethical considerations. Second, the study did not include spirometry testing, despite its known predictive value for 6MWD. ^22, 39, 56, 83, 84, 93^ Spirometry tests were not feasible due to the COVID-19 pandemic and the associated restrictions. ^97^

## Conclusion

CHB may alter sub-maximal aerobic capacity and accelerate CRMC aging, reflecting a broader phenomenon. To enhance functional capacity, a key determinant of quality-of-life, the study suggests incorporating regular physical-activity in addition to antiviral treatment. Regular physical-activity is significantly associated with a lower risk of hepatocellular carcinoma in CHB patients, ^98^ making the development of physical-activity policies and their impact on CHB populations a recommended area for future research.

## Ethical approval

This study was approved by the ethics committee of Farhat HACHED Hospital (Approval number FH/3010/2020) on August 15, 2020. All procedures in the study adhered to the ethical standards of the 1964 Helsinki Declaration.

## Informed consent

Written informed consent was obtained from all patients after receiving an explanation of the study.

## Data Availability

Zenodo: Excel data of the 54 participants (26 patients and 28 controls) included in the pilot case-control study titled “Assessment of sub-maximal aerobic capacity in North African patients with chronic hepatitis B”, https://doi.org/10.5281/zenodo.14542662. ^99^ The project contains the following underlying data:
- [Excel data of the 2 groups (26 patients and 28 controls).xlsx] (Excel file including the numerical data of the 54 participants). ^99^ Data are available under the terms of the Creative Commons Attribution 4.0 International license (CC-BY 4.0). Zenodo: Appendix 1: Methodologies and results of studies comparing 6-min walk test (6MWT) data of chronic hepatitis B (CHB) patients and healthy participants, https://doi.org/10.5281/zenodo.14584968. ^100^ The project contains the following extended data:
•
**[Appendix 1:** Methodologies and results of studies comparing 6-min walk test (6MWT) data of chronic hepatitis B (CHB) patients and healthy participants]. ^100^ Data are available under the terms of the Creative Commons Attribution 4.0 International license (CC-BY 4.0). Zenodo: STROBE checklist for ‘[Assessment of sub-maximal aerobic capacity in North African patients with chronic hepatitis B: A pilot case-control study]. https://doi.org/10.5281/zenodo.14542795. ^101^ Data are available under the terms of the Creative Commons Attribution 4.0 International license (CC-BY 4.0).
