# Supplementary material for: Assessment of sub-maximal aerobic capacity in North African patients with chronic hepatitis B: a pilot case-control study
Source: F1000Res. 2025 Apr 8;14:98. Originally published 2025 Jan 16. [Version 2] doi: 10.12688/f1000research.160390.2 (PMC12022957; doi:10.12688/f1000research.160390.2)
Supplement: Peer review history

# Reviewer report: Reviewer response for version 2

**DOI:** 10.5256/f1000research.179982.r376729

**Published:** 2025-04-23

**Authors:** Kaouther MASMOUDI

**Affiliations:** 1 University of Sfax, Sfax, Tunisia

I would like to thank the authors for the choice of this original study  which aimed to evaluate sub-maximal aerobic capacity in CHB patients compared to apparently healthy participants (control-group).

 In literature, a few articles interested in physical activity evaluation and CHB.

 The 6MWT is a valid and safe test which is largely accepted by patients and can evaluate their daily activity.

 One of the strengths of this article is the evaluation of several important indexes from simple results such 6MWD, BMI, SpO2, height, weight, sex, such as 6-min walk work (6MWW), 6MWDxBMI, 6MWDxSpO2End, estimated age (ECRMC)

 The weaknesses of this study is the lack of spirometry tests due to the COVID-19 pandemic and the associated restrictions such as explained by authors .

 Authors have considered all my comments and revised this article , particularly , they  have advanced arguments for explanation of differences between the study and control groups.

Is the work clearly and accurately presented and does it cite the current literature?

Yes

If applicable, is the statistical analysis and its interpretation appropriate?

Yes

Are all the source data underlying the results available to ensure full reproducibility?

Yes

Is the study design appropriate and is the work technically sound?

Partly

Are the conclusions drawn adequately supported by the results?

Yes

Are sufficient details of methods and analysis provided to allow replication by others?

Yes

Reviewer Expertise:

respiratory functional exploration

I confirm that I have read this submission and believe that I have an appropriate level of expertise to confirm that it is of an acceptable scientific standard.

# Reviewer report: Reviewer response for version 2

**DOI:** 10.5256/f1000research.179982.r376730

**Published:** 2025-04-14

**Authors:** Khadija Ayed

**Affiliations:** 1 Physiology and functional testing, Center of expertise in aeronautical medicine, Tunis, Tunisia; 2 PHYSIOLOGY, Faculty of medicine of Tunis, Tunis, Tunisia

I would like to thank the authors for responding to all my comments. I also thank them for all the explanations they provided to demonstrate their excellent work.

 I confirm that this is very interesting work and that it will greatly contribute to the exploration of liver disease, particularly viral hepatitis, during exercise.

 I confirm that this revised version of this manuscript meets the recommendations of the scientific editors and deserves to be accepted.

Is the work clearly and accurately presented and does it cite the current literature?

Yes

If applicable, is the statistical analysis and its interpretation appropriate?

I cannot comment. A qualified statistician is required.

Are all the source data underlying the results available to ensure full reproducibility?

Partly

Is the study design appropriate and is the work technically sound?

Yes

Are the conclusions drawn adequately supported by the results?

No

Are sufficient details of methods and analysis provided to allow replication by others?

Yes

Reviewer Expertise:

exercise physiology and pulomonary functionnal testing

I confirm that I have read this submission and believe that I have an appropriate level of expertise to confirm that it is of an acceptable scientific standard.

# Reviewer report: Reviewer response for version 1

**DOI:** 10.5256/f1000research.176281.r363571

**Published:** 2025-03-03

**Authors:** Kaouther MASMOUDI

**Affiliations:** 1 University of Sfax, Sfax, Tunisia

Thank you for this original study.

 You have clearly respond to Objectives mentioned in the introduction.

 Methodology is globally well described. 

 The only remark is the description of the method of recruitment of the control group: among health personnel, or visitor of patients or  invitation to participate via social media.

 Results:

 - The significant difference between the two groups in age, schooling level and socioeconomic level is an important factor that can influence results of six min walk test and its comparison between the two groups

 - Redondance of results between fig 2 and table 2

 - Tables 2, 3 and 4 can be regrouped in the same table.

Is the work clearly and accurately presented and does it cite the current literature?

Yes

If applicable, is the statistical analysis and its interpretation appropriate?

Yes

Are all the source data underlying the results available to ensure full reproducibility?

Yes

Is the study design appropriate and is the work technically sound?

Partly

Are the conclusions drawn adequately supported by the results?

Yes

Are sufficient details of methods and analysis provided to allow replication by others?

Yes

Reviewer Expertise:

respiratory functional exploration

I confirm that I have read this submission and believe that I have an appropriate level of expertise to confirm that it is of an acceptable scientific standard, however I have significant reservations, as outlined above.

# Response

**Published:** 2025-03-27

**Authors:** Helmi BEN SAAD

**Affiliations:** Université de Sousse, Tunisia

The authors sincerely thank the reviewer for her thorough and insightful feedback on our manuscript. We have carefully considered each comment and made the necessary revisions to address her concerns. We believe these changes have significantly improved the manuscript.

 Below are our specific responses to the reviewer's positive comments/suggestions.

**COMMENT 1**.

 .Thank you for this original study.

 .You have clearly respond to Objectives mentioned in the introduction.

**RESPONSE**

**.**We sincerely appreciate your positive feedback and recognition of our work.

 .We are pleased that the study successfully addresses the objectives outlined in the introduction.

 .Thank you for your thoughtful review.

**COMMENT 2**.

 Methodology is globally well described.

 The only remark is the description of the method of recruitment of the control group: among health personnel, or visitor of patients or invitation to participate via social media.

**RESPONSE**

 .We appreciate your valuable feedback on our methodology.

 .Regarding the recruitment of the control group, we have clarified this aspect as suggested. Additionally, we have added the following sentence to the study limitation subsection to address the potential impact of convenience sampling: “Our use of convenience sampling, a nonprobability sampling method based on the investigator's judgment, **^81^** could introduce a confounding factor. This approach may result in the underrepresentation or overrepresentation of certain groups within the sample, potentially limiting the generalizability of the findings to the broader population. **^81^** However, despite these limitations, convenience sampling remains a widely preferred method among researchers due to its affordability and ease of implementation. **^81^**.”

 .We hope this addition enhances the clarity and completeness of our methodological discussion. Thank you for your insightful review.

**COMMENT 3**

 Results: The significant difference between the two groups in age, schooling level and socioeconomic level is an important factor that can influence results of six min walk test and its comparison between the two groups

**RESPONSE**

 .Thank you for your valuable observation. We acknowledge that differences in age, schooling level, and socioeconomic status between the two groups could influence the results of the 6MWT and its comparison. We appreciate your insightful feedback, which has helped improve the clarity and robustness of our study. We have carefully considered this point in our analysis and discussion.

 .Concerning AGE, we have applied two corrective actions by using North African 6MWD reference equations to standardize 6MWD by age, and by expressing heart-rate as a percentage of maximal predicted heart-rate, accounting for age. Our corrective actions are valid approaches to account for the effect of age. For example, expressing the 6MWD as a percentage of the predicted value derived from a reference equation that includes age as an independent factor helps normalize the results across different age groups. This method adjusts for expected age-related differences in 6MWD and allows for a fairer comparison between the two groups.  However, while this adjustment reduces the direct impact of age on 6MWD, it may not completely eliminate all potential confounding effects, especially if other age-related factors (eg, motivation) influence performance. If possible, conducting additional statistical adjustments (such as ANCOVA with age as a covariate) could further refine the comparison. We have added the following paragraph: “In our study, the CG was younger than the CHB-group by 5 years. To account for this, we applied two corrective actions. We used North African 6MWD reference equations to standardize 6MWD by age, **^22, 44^** and we expressed HR as a percentage of MPHR, accounting for age. **^41^** Our corrective measures are effective strategies for accounting for the impact of age. **^22, 41, 44^** For instance, expressing the 6MWD as a percentage of the predicted value—calculated using a reference equation that includes age as an independent factor—helps standardize results across different age groups. **^57^** This approach adjusts for expected age-related variations in 6MWD, enabling a more equitable comparison between the two groups. **^57^** However, while this adjustment minimizes the direct influence of age on 6MWD, it may not entirely eliminate all potential confounding effects, particularly if other age-related factors ( eg ; motivation) affect performance. **^22, 44^** It was better to perform additional statistical adjustments, such as analysis of covariance with age as a covariate, to further enhance the accuracy of the comparison”

 .Concerning the SCHOOLING LEVEL, and SOCIOECONOMIC STATUS, we have added the following paragraph: “First, the unfavorable socioeconomic-level among our NC-CHB patients reflects findings in African CHB patient. **^77^** Second, compared to the CG, the CHB-group had higher percentages of participants with low schooling-level, and unfavorable socioeconomic-level (Table 1). On the one hand, the schooling-level was highlighted to contribute slightly but significantly to the variability of the 6MWD, accounting for an additional 2.2% of its variance. ²² In the study by Masmoudi et al., **⁵⁷** it was observed that "the higher the schooling-level was, the longer the 6MWD was". However, in an American study, ⁵⁶ a high schooling-level was identified as a non-significant independent predictor of 6MWD. On the other hand, socioeconomic-level was also recognized as a factor that slightly but significantly influenced 6MWD variability, explaining an additional 0.2–1.5% of its variance. ²² In the study by Masmoudi et al., ⁵⁷ urban participants demonstrated a significantly higher 6MWD compared to their rural counterparts.

**COMMENT 4**.

 Redundancy of results between fig 2 and table 2

**RESPONSE**

 .We agree with the reviewer, and we have deleted figure 2.

**COMMENT 5**.

 - Tables 2, 3 and 4 can be regrouped in the same table.

**RESPONSE**

****.Thank you for your suggestion regarding Tables 2, 3, and 4. We appreciate your effort to improve the clarity of the manuscript.

 .However, after your permission we would like to keep each table separate to maintain clarity and readability.

 .We hope this explanation is satisfactory and appreciate your understanding.

**COMMENT 6**.

 Is the work clearly and accurately presented and does it cite the current literature? Yes

 Is the study design appropriate and is the work technically sound? Partly

 Are sufficient details of methods and analysis provided to allow replication by others? Yes

 If applicable, is the statistical analysis and its interpretation appropriate? Yes

 Are all the source data underlying the results available to ensure full reproducibility? Yes

 Are the conclusions drawn adequately supported by the results? Yes

**RESPONSE**

**.We sincerely thank you for your thorough review and valuable feedback.**

**.We are pleased that you found our work well-presented, appropriately cited, and methodologically sound.**

**.Regarding the study design, we have carefully addressed the necessary improvements and have implemented all the requested changes.**

**.We appreciate your insightful comments, which have helped enhance the clarity and quality of our manuscript.**

**.We have added this sentence in the ACKNOWLEDGMENTS subsection: “** **The authors would like to express their sincere gratitude to the two reviewers for their excellent feedback, which has substantially improved the quality of this work. Their insightful comments and constructive suggestions were invaluable in refining our manuscript. ^104^** **”**

# Reviewer report: Reviewer response for version 1

**DOI:** 10.5256/f1000research.176281.r363568

**Published:** 2025-02-08

**Authors:** Khadija Ayed

**Affiliations:** 1 Physiology and functional testing, Center of expertise in aeronautical medicine, Tunis, Tunisia; 2 PHYSIOLOGY, Faculty of medicine of Tunis, Tunis, Tunisia

This is a rare and original study, focusing on The assessment of sub-maximal aerobic capacity in North African patients with chronic hepatitis B (CHB) through a case-control study. This is a study that could provide a great benefit to the management of chronic diseases, but also to the importance of integrating adapted physical activity as an effective and promising therapeutic means in these diseases. However, this scientific work requires a review at certain points :

**In the introduction :**

***Page 2*** *: Some studies have reported the harmful impacts of CHB on the main elements of the chain involved during adaptation to both maximal (eg; oxygen consumption ( _VO2)) and sub-maximal (eg; 6-min walk test (6MWT)) aerobic exercise…..*

- This example is not suitable, it is the **maximal oxygen consumption (V'O2 max)** which rather evaluates the tolerance to maximum effort

**Page 3** *: Exercise tolerance is commonly quantified through the measurement of _VO2 during a cardiorespiratory test* (à **cardiopulmonary exercise testing CPET)**

**Page 3** : *(On the one hand, _VO2 at peak of exercise ( _VO2peak) was a predictor of mortality, as patients with a low _VO2peak (ie; < 17 ml/kg) had a survival rate of 55%,10 and it is significantly correlated with maximal inspiratory pressure (r = 0.64) and with the Model for End-Stage Liver Disease (r = 0.91).9 On the other hand, the authors of the Saudi study reported that compared to healthy participants (n=45), patients with CHB (n=49) had a significantly lower 6-min walk distance (6MWD) by 31 m.11 The Saudi study had some methodological weaknesses that can “slightly” modify the findings.11 First, the inclusion of patients with diverse liver diseases (eg; non-cirrhotic chronic hepatitis B (NC-CHB) or C, cirrhotic), is a source of ‘perplexity’ since the clinical outcomes are different.6 Second, the absence of sample size determination is a statistical flaw.21 Third, the expression of the main outcome (ie; 6MWD) only in absolute value, and the lack of its standardization according to participants’ characteristics (eg; sex and anthropometric data), could lead to misinterpretation.8,10,11  The standardized 6MWD allows a more objective comparison between the diverse groups.22 Fourth, the use on the quantitative significance approach with a “p value” < 0.05 is criticized, and the qualitative significance approach is recommended in medical exercise research.23)*

- This whole part should be mentioned in the discussion and not in the introduction

**In the methods**

**Page 4** : *To the finest of the authors’ knowledge, no previous study has explored the incapacity* ( **aerobic capacity)** *via the 6MWT in a homogeneous group of NC-CHB patients….*

**Page 5** : *co-morbidities such as respiratory or cardiovascular diseases, systemic conditions that could influence blood test results like diabetes mellitus or renal failure, consumption of alcohol….*

-  Why did you exclude diabetics from this study knowing that diabetes does not contraindicate 6MWT?

**Page 6** : (non-smoker: <5 pack-years; smoker3: 5 pack-years **or over** )

- There is a mistake in the formula for the predicted value of the 6-minute walking distance for subjects under 40 years old

For patients under 40 years of age, you used the following formula:

 6MWD (m) = 800.05-64.71 x Sex **(0: Man; 1: Woman)** - 10.23 x BMI (kg/m2) - 1.63 x Age (years) + 2.05 x Weight (kg)

 But, according to reference 43 The formula which allows the theoretical DM6 to be calculated is as follows:

 6MWD (m) = 800.05 + 64.71 x Sex **(men:1/women:0) **- 10.23 x BMI (kg/m2) - 1.63 x Age (years) + 2.05 x Weight (kg).

 So you need to correct your formula and precise coefficient for gender.

**Page 7 :** For the ECRMC, reference 45 only contains the formula for calculating this parameter in patients over 40 years old, so it is necessary to specify the reference of the other formula which allows the calculation of the ECRMC for patients under 40 years old.

**In the Statistical analysis:**

 In this study it was necessary to calculate the Odds ratio to confirm the risk of hepatitis on aerobic capacity and the acceleration of aging of the cardiorespiratory and muscular chain”Results

**Page 7 :**

*Out of the 128 participants assessed, data from 54 participants [26 cases (15 m/11w) and 28 controls (15m/13w)] were retained for the final dataset…*

**Table 1, 2, 3 and 4 :** mean change should be mentioned for each group and the change in the measured value should be expressed as a percentage change from the baseline value (mean % change CHB Group and mean % change CG) and in this case the significance of the difference between the percentage change of the parameter for each group should be sought.

 Then, the comparison between the percentages of change will allow to evaluate the effect of the groups in the statistical analysis

**In the Discussion :**

 Discussion of methodology should come before discussion of results

 Why you did not evaluate the physiological cost index of walking (ICP). This index is defined by the difference in heart rate at the end of the 6MWT and that at rest reported to the walking speed during the 6MWT. It is an index that was created and validated by J. MacGregor and which evaluates the cost of cardiac work deployed in walking and which could have interesting correlations with the ECRMC.

 You justified the use of 6MWW by the fact that it has strong correlations with peak VO2 and this is very interesting in the absence of a technical platform allowing the measurement of peak VO2. However, the strong and significant correlations between DM6 and SpO2 are still known and validated in the literature, thus, the use of the multiplication of 6MWD by the SpO2End is useless in this work especially since you have not shown in this work correlations between this parameter and other measured parameters which could justify its usefulness.

 The same remarks for the “6MWDxBMI”, “6MWDxmuscle-mass” and “6MWDxbody-fat” especially given the lack of references and scientific validity for these parameters even in your study.

 The English version of this work leaves something to be desired.

**The conclusion :**

 The conclusion should be better structured, it should respond to the objectives of the study in a clear and precise manner and not contain references.

Is the work clearly and accurately presented and does it cite the current literature?

Yes

If applicable, is the statistical analysis and its interpretation appropriate?

I cannot comment. A qualified statistician is required.

Are all the source data underlying the results available to ensure full reproducibility?

Partly

Is the study design appropriate and is the work technically sound?

Yes

Are the conclusions drawn adequately supported by the results?

No

Are sufficient details of methods and analysis provided to allow replication by others?

Yes

Reviewer Expertise:

exercise physiology and pulomonary functionnal testing

I confirm that I have read this submission and believe that I have an appropriate level of expertise to confirm that it is of an acceptable scientific standard, however I have significant reservations, as outlined above.

# Response

**Published:** 2025-03-27

**Authors:** Helmi BEN SAAD

**Affiliations:** Université de Sousse, Tunisia

The authors sincerely thank the reviewer for her thorough and insightful feedback on our manuscript. We have carefully considered each comment and made the necessary revisions to address her concerns. We believe these changes have significantly improved the manuscript. Below are our specific responses to the reviewer positive comments/suggestions.

**COMMENT 1**.

 This is a rare and original study, focusing on the assessment of sub-maximal aerobic capacity in North African patients with chronic hepatitis B (CHB) through a case-control study. This is a study that could provide a great benefit to the management of chronic diseases, but also to the importance of integrating adapted physical activity as an effective and promising therapeutic means in these diseases.

**RESPONSE**

             Thank you so much for your kind and thoughtful comments. We are grateful for your recognition of the originality and potential impact of our study. We fully agree that integrating adapted physical activity can offer significant therapeutic benefits for patients with chronic diseases like chronic hepatitis B, and we hope our work contributes meaningfully to the ongoing discussions in this field. Your encouragement is highly appreciated.

**COMMENT 2**.

 In the introduction: Page 2 : Some studies have reported the harmful impacts of CHB on the main elements of the chain involved during adaptation to both maximal (eg; oxygen consumption ( _VO2)) and sub-maximal (eg; 6-min walk test (6MWT)) aerobic exercise…..

 This example is not suitable, it is the maximal oxygen consumption (V'O2 max) which rather evaluates the tolerance to maximum effort

**RESPONSE**

****.We agree with the reviewer.

             .The sentence was changed as: “Some studies have reported the harmful impacts of CHB on the main elements of the chain involved during adaptation to both maximal effort ( eg ; maximal oxygen consumption ( V̇O2max)) ^9, 10^ and sub-maximal aerobic exercise ( eg ; 6-min walk test (6MWT)) ^10, 11^^”^

**COMMENT 3**

 In the introduction: Page 3: Exercise tolerance is commonly quantified through the measurement of _VO2 during a cardiorespiratory test (à cardiopulmonary exercise testing CPET)

**RESPONSE**

****.We agree with the reviewer.

             .The sentence was changed as: “Exercise tolerance is commonly quantified through the measurement of oxygen consumption (V̇O2) during a cardiopulmonary exercise testing,”

**COMMENT 4**

 In the introduction: Page 3: (On the one hand, _VO2 at peak of exercise ( _VO2peak) was a predictor of mortality, as patients with a low _VO2peak (ie; < 17 ml/kg) had a survival rate of 55%,10 and it is significantly correlated with maximal inspiratory pressure (r = 0.64) and with the Model for End-Stage Liver Disease (r = 0.91).9 On the other hand, the authors of the Saudi study reported that compared to healthy participants (n=45), patients with CHB (n=49) had a significantly lower 6-min walk distance (6MWD) by 31 m.11 The Saudi study had some methodological weaknesses that can “slightly” modify the findings.11 First, the inclusion of patients with diverse liver diseases (eg; non-cirrhotic chronic hepatitis B (NC-CHB) or C, cirrhotic), is a source of ‘perplexity’ since the clinical outcomes are different.6 Second, the absence of sample size determination is a statistical flaw.21 Third, the expression of the main outcome (ie; 6MWD) only in absolute value, and the lack of its standardization according to participants’ characteristics (eg; sex and anthropometric data), could lead to misinterpretation.8,10,11  The standardized 6MWD allows a more objective comparison between the diverse groups.22 Fourth, the use on the quantitative significance approach with a “p value” < 0.05 is criticized, and the qualitative significance approach is recommended in medical exercise research.23)

 This whole part should be mentioned in the discussion and not in the introduction

**RESPONSE**

 .Thank you for your valuable feedback. We understand your suggestion to move the long paragraph from the introduction to the discussion.

 .However, we believe that this would complicate the comprehension of the introduction, as this paragraph represents the core of our introduction. In this part of the introduction, we addressed certain methodological weaknesses observed in a Saudi study (eg; inclusion of patients with diverse liver diseases, absence of sample size determination, expression of the 6MWD only in absolute value, and the lack of its standardization according to participants’ characteristics, use on the quantitative significance approach with a “p value” < 0.05), which were important, and can modify the Saudi authors’ findings. By numbering these limitations, we aim to highlight the originality and unique aspects of our study and inform readers that we have avoided some confusion factors noted in the Saudi study.

 .We hope that this explanation clarifies our reasoning, and we appreciate your understanding.

**COMMENT 5**.

 In the methods: Page 4: To the finest of the authors’ knowledge, no previous study has explored the incapacity (aerobic capacity) via the 6MWT in a homogeneous group of NC-CHB patients….

**RESPONSE**

 .We agree with the reviewer.

             .The sentence was changed as: “To the finest of the authors’ knowledge, no previous study has explored the aerobic incapacity via the 6MWT in a homogeneous group of NC-CHB patients compared to a control-group (CG) of “apparently” healthy participants. The main aim of this case-control study was to compare the 6MWT data of the CHB-group and CG. The null hypothesis was that the two groups would have a comparable 6MWD ( ie ; the main outcome).”

**COMMENT 6**.

 In the methods Page 5: co-morbidities such as respiratory or cardiovascular diseases, systemic conditions that could influence blood test results like diabetes mellitus or renal failure, consumption of alcohol….

  Why did you exclude diabetics from this study knowing that diabetes does not contraindicate 6MWT?

**RESPONSE**

 .Thank you for this remark.

 .A previous Tunisian study (Latiri I, et al.: Six-minute walk test in non-insulin-dependent diabetes mellitus patients living in Northwest Africa. *Diabetes Metab. Syndr. Obes.* 2012;5:227–245) concluded that diabetes mellitus seems to accelerate the decline of the submaximal aerobic capacity evaluated through the 6MWD. For that reason, we have excluded diabetics from our study.

 .We have added the aforementioned reference inside the text after diabetes mellitus (diabetes mellitus ^25^)

**COMMENT 7**.

 In the methods Page 6: (non-smoker: <5 pack-years; smoker3: 5 pack-years or over )

**RESPONSE**

 .Thank you for this remark.

 .Correction done inside the paper: ³5 pack-years

**COMMENT 8**.

 There is a mistake in the formula for the predicted value of the 6-minute walking distance for subjects under 40 years old

 For patients under 40 years of age, you used the following formula:

 6MWD (m) = 800.05-64.71 x Sex (0: Man; 1: Woman) - 10.23 x BMI (kg/m2) - 1.63 x Age (years) + 2.05 x Weight (kg)

 But, according to reference 43 The formula which allows the theoretical DM6 to be calculated is as follows:

 6MWD (m) = 800.05 + 64.71 x Sex (men:1/women:0) - 10.23 x BMI (kg/m2) - 1.63 x Age (years) + 2.05 x Weight (kg).

 So you need to correct your formula and precise coefficient for gender.

**RESPONSE**

 .Thank you for your remark.

 .Sorry to let you know that there is no mistake in the formula for the predicted value of the 6MWD for subjects under 40 years old. We have simply have simply inverted codes for sex [ie; (0: Man; 1: Woman) by (men:1/women:0)] and therefore we have changed “-64.71 x Sex” by “+ 64.71 x Sex”. We have changed the signs in order to make the formulas of subjects aged more and less than 40 years homogenized.

 .Therefore, our 6MWD results are correct. Thank you for your comprehension.

**COMMENT 9**.

 In the methods Page 7: For the ECRMC, reference 45 only contains the formula for calculating this parameter in patients over 40 years old, so it is necessary to specify the reference of the other formula which allows the calculation of the ECRMC for patients under 40 years old.

**RESPONSE**

 .Thank you for this remark.

 .Correction done inside the paper.

**COMMENT 10**.

 In the Statistical analysis:

 In this study, it was necessary to calculate the Odds ratio to confirm the risk of hepatitis on aerobic capacity and the acceleration of aging of the cardiorespiratory and muscular chain.

**RESPONSE**

****.Thank you for your remark.

****.We have calculated the odds ratio (OR) to confirm the risk of hepatitis on aerobic capacity and the acceleration of aging of the cardiorespiratory and muscular chain. Mainly we have calculated the OR for the following two data: abnormal 6MWD and Low HR (we do not calculated the OR for Desaturation and High dyspnea _End_ since no participant from both groups exhibited Desaturation or High dyspnea _End_ (see Table 4).

 .We have added the following sentences inside the paper:

*Abstract:

 “The CHB-group was 8.1 and 14.3 times more likely to have chronotropic insufficiency and abnormal 6MWD than the CG, respectively”

*Manuscript:

 “Moreover, to confirm the risk of CHB on aerobic capacity, odds ratios were calculated for abnormal 6MWD and chronotropic insufficiency”.

 “The CHB-group was 8.1 times more likely to have chronotropic insufficiency than the CG.”

 “The CHB-group was 14.3 times more likely to have an abnormal 6MWD than the CG.”

**COMMENT 11**.

 ”Results Page 7: Out of the 128 participants assessed, data from 54 participants [26 cases (15 m/11w) and 28 controls (15m/13w)] were retained for the final dataset…

**RESPONSE**

 .Thank you for this remark.

 .Correction done inside the paper: “Out of the 128 participants assessed, data from 54 participants [26 cases (15 men/11 women) and 28 controls (15 men/13 women)] were retained for the final dataset.”

**COMMENT 12**.

 ”Results Page 7: Table 1, 2, 3 and 4 : mean change should be mentioned for each group and the change in the measured value should be expressed as a percentage change from the baseline value (mean % change CHB Group and mean % change CG) and in this case the significance of the difference between the percentage change of the parameter for each group should be sought. Then, the comparison between the percentages of change will allow to evaluate the effect of the groups in the statistical analysis.

**RESPONSE**

****.We failed to correctly understand your comment.

****.Inside the statistical analysis subsection, we have added the following sentence: For quantitative data, mean percentage changes (%) between the two groups were calculated [mean percentage change = 100 x (CHB-group mean value minus CG mean value)/CHB-group mean]. For each group, percentage delta changes (∆) between data determined at _Rest _and _End_ walk were calculated for HR, SpO _2_, SBP, DBP, and dyspnea VAS [ ie ; ∆HR (%) = 100 x (HR _End_ – HR _Rest_)/HR _Rest_, ∆SpO _2_ (%) = 100 x (SpO _2End_ - SpO _2Rest_)/ SpO _2Rest_, ∆SBP (%) = 100 x (SBP _End_ – SBP _Rest_)/SBP _Rest_, and ∆DBP (%) = 100 x (DBP _End_ – DBP _Rest_)/DBP _Rest_].

             .We have not calculated percentage delta changes (∆) for Dyspnea VAS, since all _Rest _values were equal to zero.

 .In tables 1, 2, and 3, mean changes were calculated between the two groups for quantitative data. We cannot calculate mean percentage changes for each group because there is no baseline and after intervention data. We have applied all needed changes in Table 1.

             .In tables 3 and 4, we have calculated, for each group, the change (∆) in HR, SBP, DBP, and VAS. Then we have compared the changes between the two groups.

             .Please consult all changes inside the abstract, text and tables.

**COMMENT 13**.

 In the Discussion: Discussion of methodology should come before discussion of results

**RESPONSE**

****.Thank you for your suggestion. We understand that there is two approaches related to the subjects you raised: should we begin with the discussion of results or methods?

 .In an original medical article, it is generally recommended to begin the Discussion section by interpreting and discussing the key results of the study. This approach helps maintain a logical flow, as readers are usually most interested in understanding the study’s findings before delving into methodological considerations.

 .Thank you for your comprehension.

**COMMENT 14**.

 In the Discussion: Why you did not evaluate the physiological cost index of walking (ICP). This index is defined by the difference in heart rate at the end of the 6MWT and that at rest reported to the walking speed during the 6MWT. It is an index that was created and validated by J. MacGregor and which evaluates the cost of cardiac work deployed in walking and which could have interesting correlations with the ECRMC.

**RESPONSE**

****.Thank you for your remark concerning the physiological cost index (PCI).

 .We have consulted the paper by MacGregor J ‘The evaluation of patient performance using long-term ambulatory monitoring technique in the domiciliary environment. Physiotherapy. 1981 Feb 10;67(2):30-3. PMID: 7312961”.

             .The PCI, created and validated by J. MacGregor, is a metric used to quantify the energy cost or efficiency of walking, particularly in individuals with mobility impairments or disabilities. It is designed to provide a standardized way to assess walking performance by considering factors such as speed, energy expenditure, and physiological effort. The PCI is often used in clinical and rehabilitation settings to evaluate the effectiveness of interventions, such as prosthetics, orthotics, or physical therapy, on walking ability. This was not the aim of our study.

 .The PCI ***i)*** measures the energy cost of walking, typically expressed as the amount of energy expended per unit distance walked ( ***e.g.***, oxygen consumption per meter); ***ii)*** often normalizes energy expenditure to body weight or other physiological parameters to allow for comparisons across individuals, ***iii)*** is useful for identifying inefficiencies in walking patterns and guiding interventions to improve mobility. Again, this was not the main aim of our study.

             .The PCI is calculated using data from walking tests, often involving measures of: ***i)*** Oxygen consumption during walking, ***ii)*** Walking speed (distance over time), and ***iii)*** Body weight or other anthropometric data. Its formula typically involves dividing the energy expenditure ( ***e.g.***, oxygen consumption) by the product of walking speed and body weight, though the exact formula may vary depending on the study or application. In our study, we cannot calculate the PCI, since we have not all needed data.

             .In practice, the PCI can be calculated as PCI (bpm*min/m) = (HR _End_ (bpm) minus HR _Rest_) (bpm)/walk speed (m/min). Mean PCI values for healthy adults have been reported to be between 0.23 and 0.42 (MacGregor J. The evaluation of patient performance using long-term ambulatory monitoring technique in the domiciliary environment. Physiotherapy. 1981 Feb 10;67(2):30-3. PMID: 7312961. 13-15; Graham RC, Smith NM, White CM. The reliability and validity of the physiological cost index in healthy subjects while walking on 2 different tracks. Arch Phys Med Rehabil. 2005 Oct;86(10):2041-6. doi: 10.1016/j.apmr.2005.04.022. PMID: 16213251). In this case we need to calculate the walk speed by dividing the 6MWD by 6.

             .Since we have previously published our protocol without reporting our intention to evaluate the PCI (see: Bergaoui J, Latiri I, Ben Saad H. Deficiency, incapacity and social disadvantage of patients with chronic hepatitis B: a case-control study. Tunis Med. 2021 Juillet;99(7):682-692. PMID: 35260999; PMCID: PMC8796680. ), at this stage we cannot add this interesting variable. However, in the revised version, we have recommended to evaluate the PCI in future studies. The following sentence was added: “In future research, it would be valuable to assess the physiological cost index. **^97, 98^** The latter (expressed as heartbeats per meter), determined by dividing the difference between ending and resting HRs by walking speed, represents the additional HR demand during walking. **^97, 98^** Studies have shown that healthy adults typically exhibit mean physiological cost index values ranging from 0.23 to 0.42. **^97, 98^**”

**COMMENT 15**.

 In the Discussion : You justified the use of 6MWW by the fact that it has strong correlations with peak VO2 and this is very interesting in the absence of a technical platform allowing the measurement of peak VO2. However, the strong and significant correlations between DM6 and SpO2 are still known and validated in the literature, thus, the use of the multiplication of 6MWD by the SpO2End is useless in this work especially since you have not shown in this work correlations between this parameter and other measured parameters, which could justify its usefulness.

 In the Discussion :The same remarks for the “6MWDxBMI”, “6MWDxmuscle-mass” and “6MWDxbody-fat” especially given the lack of references and scientific validity for these parameters even in your study.

**RESPONSE**

****.Thank you for your remark. We understand your point of view concerning the indices “6MWD x SpO _2End_”, “6MWD x BMI”, “6MWD x MM” and “6MWD x BF”.

             .In order to shorten our paper, we have deleted all these indices.

             .Thank you for this interesting remark.

**COMMENT 17**.

 The English version of this work leaves something to be desired.

**RESPONSE**

****.Thank you for your pertinent remark.

             .We have revised the English version. We have used a generative AI tool for that reason.

 .Inside the paper, we have made the following declaration related to the use of AI chatbots: “In preparing this paper, the authors used ChatGPT ephemeral, to revise some passages of the manuscript, to double-check for any grammar mistakes or improve academic English only. After using this tool, the authors have reviewed and edited the content as necessary and take full responsibility for the content of the publication.”

**COMMENT 18**.

 The conclusion: The conclusion should be better structured, it should respond to the objectives of the study in a clear and precise manner and not contain references.

**RESPONSE**

****.Thank you for your valuable feedback and insightful comments. We appreciate your suggestion regarding the conclusion.

 .We have revised the conclusion to ensure a clearer and more structured response to the study’s objectives, while also removing any references. Your input is highly valuable in strengthening the clarity and impact of the manuscript.

 .Here is the new conclusion: “Our study reveals that NC-CHB impairs sub-maximal aerobic capacity, with patients showing reduced 6MWD and 6MWW compared to controls. Chronotropic insufficiency and accelerated cardiorespiratory-muscular aging suggest systemic dysfunction beyond liver disease. Our study contributes to the limited literature on CHB-related functional impairment and calls for integrated management strategies addressing both hepatic and extrahepatic manifestations of the disease. Future research should explore mechanisms and interventions to address these impairments.”

**COMMENT 19**.

 Is the work clearly and accurately presented and does it cite the current literature?

 Yes

 Is the study design appropriate and is the work technically sound?

 Yes

 Are sufficient details of methods and analysis provided to allow replication by others?

 Yes

 If applicable, is the statistical analysis and its interpretation appropriate?

 I cannot comment. A qualified statistician is required.

 Are all the source data underlying the results available to ensure full reproducibility?

 Partly

 Are the conclusions drawn adequately supported by the results?

 No

**RESPONSE**

 .Thank you for your thorough review and valuable feedback.

 .We appreciate your positive assessment of the clarity, study design, and methodology.

 .Regarding your comment on statistical analysis, we confirm that all our statistics are correct.

 .Additionally, we have addressed the issue of data availability to enhance reproducibility by uploading an Excel data of the 54 participants (26 patients and 28 controls) included in the pilot case-control study titled. Please see (reference 100): https://doi.org/10.5281/zenodo.14542662.

 .Finally, we have revised the conclusion to ensure that it is fully supported by the results.

 .Your insights are greatly appreciated and helped improve the quality of the manuscript.

 .We have added this sentence in the ACKNOWLEDGMENTS subsection: “The authors would like to express their sincere gratitude to the two reviewers for their excellent feedback, which has substantially improved the quality of this work. Their insightful comments and constructive suggestions were invaluable in refining our manuscript. ^104^”
